# Southern Hemisphere Influenza and Vaccine Effectiveness Research and Surveillance

**DOI:** 10.1111/irv.12315

**Published:** 2015-06-09

**Authors:** Qiu Sue Huang, Nikki Turner, Michael G Baker, Deborah A Williamson, Conroy Wong, Richard Webby, Marc-Alain Widdowson

**Affiliations:** a Institute of Environmental Science and ResearchWellington, New Zealand; b University of AucklandAuckland, New Zealand; c University of OtagoWellington, New Zealand; d Auckland District Health BoardAuckland, New Zealand; e Counties Manakau District Health BoardAuckland, New Zealand; f WHO Collaborating Centre, St Jude Children's Research HospitalMemphis, TN, USA; g Centers for Disease Control and Prevention (CDC)Atlanta, GA, USA

**Keywords:** disease burden, epidemiology, immunology, influenza, risk factors, vaccine effectiveness

## Abstract

The 2009 influenza A(H1N1)pdm09 pandemic highlighted the need for improved scientific knowledge to support better pandemic preparedness and seasonal influenza control. The Southern Hemisphere Influenza and Vaccine Effectiveness Research and Surveillance (SHIVERS) project, a 5-year (2012–2016) multiagency and multidisciplinary collaboration, aimed to measure disease burden, epidemiology, aetiology, risk factors, immunology, effectiveness of vaccination and other prevention strategies for influenza and other respiratory infectious diseases of public health importance. Two active, prospective, population-based surveillance systems were established for monitoring influenza and other respiratory pathogens among those hospitalized patients with acute respiratory illness and those enrolled patients seeking consultations at sentinel general practices. In 2015, a sero-epidemiological study will use a sample of patients from the same practices. These data will provide a full picture of the disease burden and risk factors from asymptomatic infections to severe hospitalized disease and deaths and related economic burden. The results during the first 2 years (2012–2013) provided scientific evidence to (a) support a change to NZ's vaccination policy for young children due to high influenza hospitalizations in these children; (b) contribute to the revision of the World Health Organization's case definition for severe acute respiratory illness for global influenza surveillance; and (c) contribute in part to vaccine strain selection using vaccine effectiveness assessment in the prevention of influenza-related consultations and hospitalizations. In summary, SHIVERS provides valuable international platforms for supporting seasonal influenza control and pandemic preparedness, and responding to other emerging/endemic respiratory-related infections.

## Background

The 2009 influenza A(H1N1)pdm09 pandemic provided a test of global preparedness to assess the epidemiology of a pandemic and to respond appropriately and rapidly. The world was ill-prepared to respond to a severe influenza pandemic or to any similarly global, sustained and threatening public health emergency.^1^ One fundamental constraint highlighted during the pandemic was the limited understanding of the epidemiology and severity of the pandemic which in turn hampered international efforts to mount an appropriate response.^2^ Rapid assessment of the epidemiologic, virologic and clinic features of a pandemic is essential to provide critical information to decision-makers on how to minimize morbidity and mortality, and mitigate potential economic and societal disruption.

Soon after the pandemic virus emerged in April 2009 in Mexico and spread globally, public health leaders, anxious to understand the full breadth of influenza epidemiology, turned their attention to countries in southern temperate areas with an upcoming influenza season. This demonstrated the absence of an established real-time system in the Southern Hemisphere to provide more complete surveillance of an influenza pandemic. Also, such a system would help monitor the epidemiology of new strains of seasonal influenza and the effectiveness of vaccination, both for the Southern Hemisphere and for upcoming Northern Hemisphere seasons.

In December 2010, the US Centers for Disease Control and Prevention (US-CDC) made a funding opportunity announcement for a temperate Southern Hemisphere site to conduct research on the disease burden, epidemiology, and prevention of influenza and other respiratory diseases of public health importance.

New Zealand (NZ) is a temperate Southern Hemisphere country with a population of 4·4 million people. The influenza season mainly occurs from June to September.^3^–^6^ NZ's predominantly public-funded healthcare system with associated integrated health information systems is a strong asset in conducting population-based research. All New Zealanders are assigned a unique health identifier allowing tracking of healthcare utilization over time and confidential record linkage to multiple databases including hospitalization and surveillance data. Additionally, patients are registered with primary care providers who maintain highly computerized records with detailed demography, immunization status and clinical information. The NZ population is well characterized in terms of demographic structure, particularly by ethnicity and socio-economic status. Indigenous Maori and Pacific peoples (collectively about 20% of the population) appear particularly vulnerable to influenza and other respiratory infections.^3^,^7^

In October 2011, led by the Institute of Environmental Science and Research (ESR), a multiagency and multidisciplinary project ‘Southern Hemisphere Influenza and Vaccine Effectiveness Research and Surveillance’ (SHIVERS) was established for a 5-year period (2012–2016) as a result of the award received from US-CDC. This collaboration is between ESR, Auckland District Health Board (ADHB), Counties Manukau District Health Board (CMDHB), University of Auckland, University of Otago, WHO Collaborating Centre at St Jude Children's Research Hospital and US-CDC.

In this article, we describe the objectives and study designs of SHIVERS. We also describe lessons learned from the first 2 years and planned future studies as well as international applications.

## Aim and objectives

The overarching aim of SHIVERS is to comprehensively investigate the disease burden, epidemiology, aetiology, risk factors, immunology, effectiveness of vaccination and other prevention strategies for influenza and other respiratory viral diseases of public health importance.

The project consists of 9 objectives as detailed in Table1. They can be divided into five main streams:

**Table 1:** Nine objectives of SHIVERS

Objectives	Specific aims	Methods
Obj 1 (Primary): Understand severe respiratory diseases	Measure incidence, prevalence, risk factors, clinical outcomes and severity for hospitalized severe acute respiratory illness (SARI) and associated influenza and other respiratory infections as well as understand influenza contribution to patients not meeting SARI case definition	Active, prospective, continuous, population-based surveillance for influenza and other respiratory pathogens among hospitalized patients with respiratory illness.
Obj 2 (Primary): Assess influenza vaccine effectiveness	Assess the annual effectiveness of seasonal trivalent inactivated influenza vaccine (TIV) in preventing general practice visits and hospitalizations for laboratory-confirmed influenza	Using case test-negative control design
Obj 3: Investigate interaction between influenza and other pathogens	Investigate the potential role of pathogen co-infections (viral–viral, viral–bacteria) in patient outcome, severity, aetiology, demography and underlying risk conditions.	Simultaneous testing by real-time RT-PCR assays for 8 respiratory viruses for all SARI and ILI patients. Simultaneous testing for respiratory virus and bacteria by blood culture, urinary antigen test and PCR for some SARI cases and non-SARI patients.
Obj 4: Understand aetiologies and causes of respiratory mortality	Real-time monitoring all SARI in-hospital deaths and the associated aetiologies	The same methods as objectives 1 and 3
Obj 5: Understand non-severe respiratory diseases	Measure incidence, prevalence, risk factors, clinical spectrums for consultation-seeking influenza-like illness (ILI) and associated influenza and other respiratory infections	Active, prospective, population-based surveillance for influenza and other respiratory pathogens among persons enrolled in sentinel general practices who seek medical consultations.
Obj 6: Estimate influenza infection via serosurvey	Estimate annual incidence of infection and identify potential risk factors for infection with seasonal influenza among different age and ethnic groups	Conducting a serologic cohort study using sentinel general practices recruited for Objective 5
Obj 7: Identify and quantify risk factors for getting influenza	Risk factors include host, socio-economic, underlying medical conditions, health intervention, health service utilization, and environmental and behavioural factors	Using well-characterized socio-demographic distribution data and use case-control design with several comparison/control groups
Obj 8: Assess immune response in severe, moderate influenza cases, related risk groups and individuals with serologically defined influenza infection	Study humoral and cellular immunologic responses in a subset of SARI and ILI patients and risk groups with confirmed influenza and individuals with serologically defined influenza infection.	Measure antihemagglutinin (HA) antibodies, antineuraminidase (NA) antibodies, isotypes of responding antibodies, influenza-specific CD4+, CD8+ T cells, surface markers and key cytokines expression levels
Obj 9: Estimate healthcare, societal economic burden caused by influenza and vaccine cost-effectiveness	Estimate influenza-associated healthcare and societal economic burden and vaccine cost-effectiveness among a range of different subpopulations	Estimate direct medical costs and indirect societal cost (e.g. loss of productivity, loss of earning and loss of life) for the study population and subpopulations

### Burden and epidemiology

Influenza disease burden data are essential to allocate limited health resources, assist influenza vaccination policy development and improve vaccine uptake, particularly for subpopulations at risk. However, the evidence to support valid and precise estimates of influenza disease burden globally remains weak with low quality, partly due to the short duration of studies and the heterogeneity of study settings and methods (statistical modelling, active versus passive case findings, virological versus clinical detection).^8^–^14^ In addition, there is scarce information on sero-epidemiologic investigation of seasonal influenza at a population level. Serology can detect both symptomatic and asymptomatic infections, thus estimating the true incidence of influenza infection. This parameter cannot be determined by either disease surveillance programmes or detection of virologically confirmed cases as they would vastly underestimate influenza incidence and overestimate severity.^15^–^18^

SHIVERS allows calculation of rates of infection and different clinical presentations in the same population at the same time for an accurate picture of the relative severity of influenza infection in the population and vulnerable subpopulations at four levels: (a) severe hospitalized disease; (b) moderate disease requiring a general practice visit; (c) mild disease not requiring a general practice visit; (d) incidence of infection (symptomatic and asymptomatic).

### Aetiology

SHIVERS provides an integrated platform for the study of respiratory diseases caused by influenza and other common and emerging respiratory pathogens. The aetiological component allows us to (1) monitor antigenic drift of seasonal influenza viruses, contributing to WHO's annual vaccine strain selection; (2) support pandemic preparedness including surveillance for new subtypes of influenza A viruses (e.g. A(H7N9)); (3) monitor common non-influenza respiratory pathogens to understand their impact on the disease and epidemiology; and (4) provide early detection for emerging respiratory viruses (e.g. MERS-CoV).

There is increasing evidence in the literature for the importance of polymicrobial infections. However, there remain gaps in our understanding of respiratory virus co-detection and whether this represents co-infection and affects clinical disease manifestations and severity. There are contradictory reports with some suggesting that co-infections increase the severity of respiratory disease,^19^–^23^ while others have found either no association^24^–^28^ or that co-infections may actually be protective.^29^ Additionally, bacterial co-infections associated with cases of influenza are a leading cause of severe morbidity and mortality: bacterial co-infections complicated nearly all influenza deaths in the 1918 pandemic and up to 34% of the 2009 A(H1N1)pdm09 infections managed in intensive care units worldwide.^30^,^31^ SHIVERS will help our understanding of the potential role of pathogen co-detection in patient outcome, severity, aetiology, demography and underlying risk conditions.

### Vaccine effectiveness

Influenza vaccine strain selection requires annual consultations and frequent updates to match the antigenic drift of the circulating viral strains, and ample evidence indicates that influenza vaccine effectiveness (VE) varies not only by virus type (subtype) but also from year to year.^32^,^33^ Robust and timely vaccine effectiveness estimates are important to measure the public health benefit of seasonal influenza control strategies, pandemic preparedness and vaccine strain selection.^34^ Many VE estimates derive from observational studies with existing data collecting systems which often have multiple limitations and biases, and there are international calls for more rigorous VE studies.^34^–^39^ SHIVERS is providing robust and timely estimations of the protective effect of seasonal influenza vaccine in the prevention of hospitalizations and general practice consultations for laboratory-confirmed influenza using case test-negative control methods.^40^,^41^

### Immune response

An individual's immune response to influenza infection can vary depending on many factors (e.g. age, underlying conditions and ethnicity). Clinical observation during the 2009 pandemic indicated that the unexpected low morbidity and mortality rates in the elderly were in part due to their cross-reactive immunity.^3^,^42^,^43^ There are knowledge gaps regarding each component of adaptive immune responses in determining an individual's risk of acquiring influenza virus infection and the severity of the resulting disease: antihemagglutinin (HA) antibodies, antineuraminidase (NA) antibodies, isotypes of responding antibodies, influenza-specific CD4+, CD8+ T cells, surface markers and key cytokines.^44^,^45^ Additionally, there are scarce data on the correlation of cellular immunologic and neuraminidase targeted antibody responses in individuals with serologically (anti-HA antibodies) defined influenza infection.^46^,^47^By interconnecting the epidemiological and immune studies in severe and moderate disease cases and high-risk subgroups (e.g. Pacific and Maori ethnic groups) and individuals with serologically defined influenza infection, SHIVERS will facilitate our understanding of host immune responses that determine an individual's risk of acquiring influenza infection or developing severe disease.

### Risk factors

Identification and quantification of risk factors for influenza infection and poor outcomes (hospitalization, ICU treatment, death) provides evidence to inform decisions on targeted pharmaceutical (vaccinations, antivirals), healthcare (e.g. improved treatment of comorbidities) and non-pharmaceutical (e.g. exposure to infections) interventions to reduce the risk of seasonal and pandemic influenza. Elderly people have a significantly higher risk of influenza-associated death compared with non-elderly people.^48^ Additionally, the 2009 pandemic in NZ revealed that the risk of hospitalization and death was markedly higher for Maori and Pacific people, and those from the most deprived socioeconomic groups.^3^,^42^,^49^ However, it is not clear whether these socio-demographic factors are independent risk factors for influenza. Furthermore, some chronic health conditions (high body mass index, asthma and pregnancy) have been shown to increase the risk of having a poor outcome from influenza infection.^50^–^53^ In NZ, household crowding has been identified as a risk factor for transmission of meningococcal disease,^54^rheumatic fever^55^ and tuberculosis^56^ and may also be contributing to higher rates of influenza for some populations. The household setting (crowding, housing conditions) may influence transmission of influenza, but these effects remain poorly understood.^57^–^59^ SHIVERS will provide a multifaceted understanding of influenza risk that considers organism, host and environmental factors and opportunities for intervention. This comprehensive and quantitative approach will include detailed consideration of the independent contributions of host ethnicity, socio-economic position, chronic illness status, obesity, household environment exposures and infecting virus.

## Study designs

### Study sites

**Figure 1:**
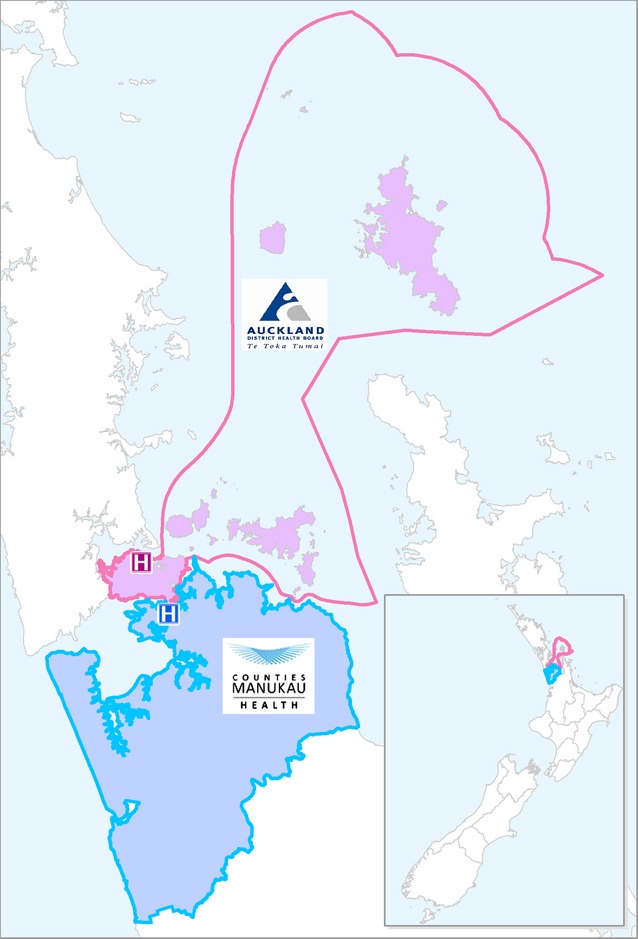
A map of New Zealand, Auckland District Health Board (ADHB) and Counties Manukau District Health Board (CMDHB).

SHIVERS study sites are located within two District Health Boards of the Auckland region of NZ: ADHB and CMDHB (Figure1). This is a predominantly urban population of 906 000 people, with a wide spectrum of socio-economic, cultural, ethnic and demographic groups broadly similar to the New Zealand population.^60^

We established two surveillance platforms (hospital and sentinel general practice) in the two DHBs:

### Hospital surveillance platform

Four publicly funded hospitals serve the secondary healthcare needs for all residents of the two DHBs: Auckland City Hospital and the associated Starship Children's Hospital (ADHB), and Middlemore Hospital and the associated Kidz First Children's Hospital (CMDHB).

**Figure 2:**
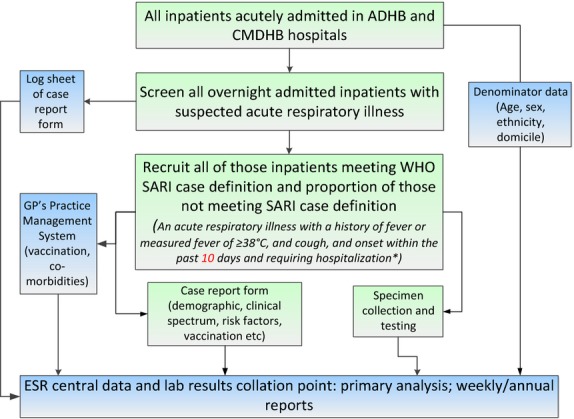
SHIVERS hospital surveillance platform.*Note: In 2012, the WHO interim SARI case definition was used (i.e. onset within the past 7 days). Since 2013, the WHO final SARI case definition was used (i.e. onset within the past 10 days).

**Figure 3:**
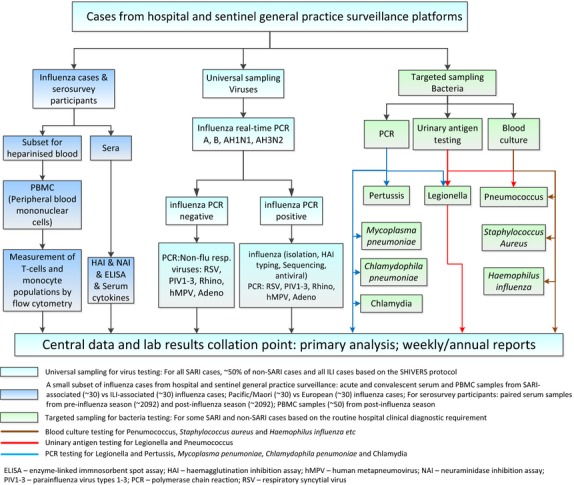
Sampling and testing for cases from hospital and sentinel general practice surveillance platforms.

In 2012, we began active, prospective, continuous, population-based surveillance for influenza and other respiratory pathogens among persons residing in the two DHBs hospitalized for respiratory disease (Figure2). Research nurses reviewed daily records of all overnight acutely admitted inpatients to identify any inpatient with a suspected acute respiratory illness (ARI). They interviewed these patients by applying the World Health Organization (WHO) interim SARI case definition: ‘an acute respiratory illness with a history of fever or measured fever of ≥38°C, and cough, and onset within the past 7 days, and requiring inpatient hospitalization’.^61^ Since 2013, the WHO final SARI case definition has been used with onset changed from ‘7 days’ to ‘10 days’. The patients were differentiated into SARI cases (those who met the SARI case definition) and non-SARI patients (those with ARI who did not meet the SARI case definition). A case report form captured information on demography, history of presenting illness, comorbidities, influenza vaccination history, disease outcome and risk factors.^60^ If a patient met the SARI case definition, a respiratory specimen (nasopharyngeal swab or aspirate) was collected and tested simultaneously for influenza and other respiratory viruses by real-time reverse transcription (RT) polymerase chain reaction (PCR) techniques: influenza virus, respiratory syncytial virus (RSV), rhinovirus, parainfluenza virus types 1–3, adenovirus and human metapneumovirus (Figure3).^60^ A systematic sample of about 50% of non-SARI patients were also interviewed and provided a respiratory sample, in addition to those from whom a specimen was collected for clinical purposes. Some SARI cases and non-SARI patients were also tested for clinical purposes for a range of respiratory bacteria (e.g. *Streptococcus pneumonia, Staphylococcus aureus, Haemophilus influenza*) by blood culture, urinary antigen test and PCR.^60^

### Sentinel general practice surveillance platform

**Figure 4:**
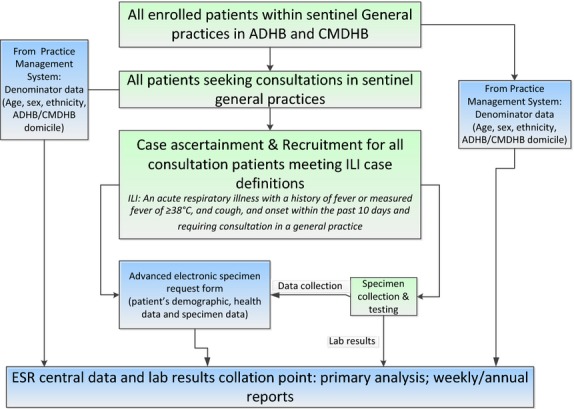
SHIVERS sentinel general practice surveillance platform.

In 2013, we began active, prospective, population-based surveillance for influenza and other respiratory pathogens among persons enrolled in sentinel general practices who seek medical consultations (Figure4). Eighteen sentinel general practices situated within ADHB and CMDHB were recruited. These practices have a combined total of 103 752 enrolled patients, covering approximately 12% of the ADHB and CMDHB population. The participating general practitioners (GP) and practice nurses (PN) assessed all consultation-seeking patients. If a patient met the influenza-like illness (ILI) case definition: ‘an acute respiratory illness with a history of fever or measured fever of ≥38°C, and cough, and onset within the past 10 days, and requiring consultation in that general practice’, a respiratory specimen (nasopharyngeal swab or throat swab) was collected to test for influenza and other respiratory viruses by real-time RT-PCR (Figure3). GP/PN documented the components of the case definition that were present and recorded patients who met the ILI case definition in an advanced electronic form designed for the practice management system (PMS). Patient information already captured in the PMS was automatically retrieved, including demography, comorbidities, vaccination history and regular medication list. Further data were captured by interviewing ILI patients regarding influenza vaccination obtained elsewhere, pregnancy and a clinician's judgement of obesity.

### Other studies based on these platforms

To more fully understand the epidemiology of influenza, these two platforms will be leveraged for other studies: (a) *sero-epidemiological study*: To obtain rates of mild influenza illness that do not trigger GP visits as well as symptomatic/asymptomatic infections, the enrolled patients in those sentinel general practices will be used to randomly select a cohort of persons (stratified by age and ethnicity) and followed through one influenza season. The serologic surveys will measure pre- and post-season antibodies to circulating influenza strains using relevant vaccine reference strains as antigens; (b) *immunology study*: A subset of samples from severe, moderate influenza cases, related risk groups and individuals with serologically defined influenza infection are selected for the study of humoral and cellular immune responses (Figure3); and (c) *remaining studies*: The combined laboratory testing results and metadata collected from these platforms are used to study vaccine effectiveness, interaction between respiratory pathogens, respiratory mortality, risk factors and economic burden and vaccine cost-effectiveness.

**Figure 5:**
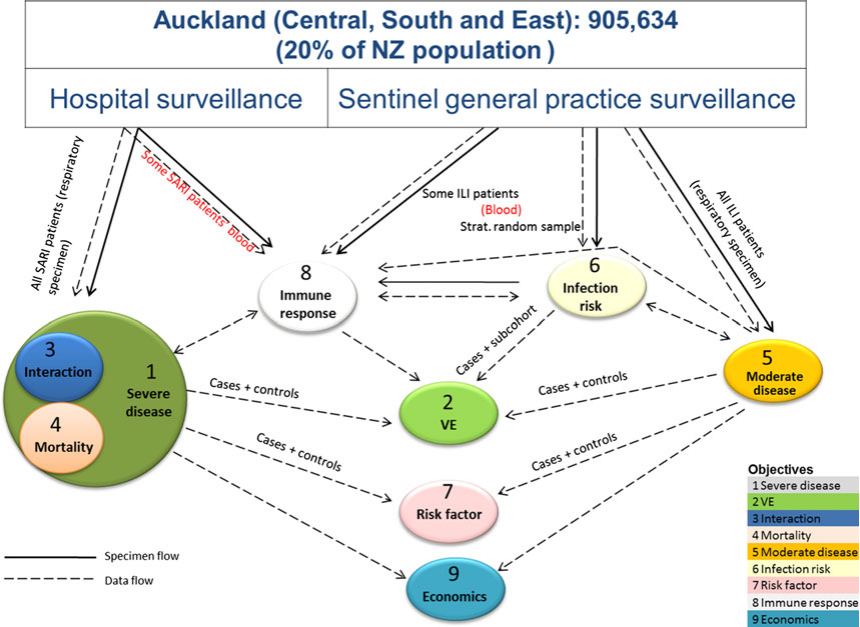
SHIVERS surveillance platforms and interconnectedness of the 9 objectives.

Our innovative study design interconnecting multiple objectives, in addition to exploiting NZ's unique healthcare structure, will maximize efficiency and study power. The two surveillance platforms provided specimens and data to serve the nine objectives of SHIVERS. Figure5 shows how each of the objectives is linked to each other and maps data and specimen flows between them.

## Lessons learned from the first 2 years and future studies in the next 3 years

### Refinement of the SARI case definition

Since its conception in 2006, SARI surveillance has become a recognized international standard for monitoring hospitalized severe respiratory disease related to influenza and other pathogens.^61^–^64^ Revisions of the SARI case definition have occurred over this time.^65^–^67^ It was designed to monitor trends in severe influenza disease and to best capture the majority of influenza respiratory disease to estimate the burden of influenza-associated respiratory hospitalizations, and risk factors for severe disease.^63^ The initial SARI case definition included the symptom onset of acute respiratory illness within 7 days.^61^

The SHIVERS results in 2012 showed that a small proportion (7%) of influenza cases had specimens collected 8–9 days after the symptom onset. These specimens only consisted of a small proportion (9%) of total specimens; thus, the cost of testing was minimal (manuscript in preparation). This result was shared with the WHO global influenza programme. It contributed to a change in the final WHO SARI case definition, with onset shifting from ‘within 7 days’ to ‘within 10 days’.^63^

### Burden and epidemiology

SHIVERS allows the estimation of influenza disease burden and risk factors at various levels of severity.

Firstly, the disease burden of severe influenza is estimated from the hospital surveillance platform. It measured population-based incidence for SARI-associated influenza hospitalizations including ICU admissions and in-hospital deaths as it provided reliable numerators and denominators, thus without a need for additional healthcare utilization surveys.^68^–^71^ Our first-year findings (30 April 2012 to 28 April 2013) showed that the SARI-associated influenza hospitalization rate was substantial with the overall adjusted annual incidence of 54/100 000 persons (manuscript in preparation). This rate was similar to US data on influenza-associated hospitalizations during 1979–2001, with an average annual incidence of 36·8/100 000.^12^ The very young (0–4 years) and elderly (≥65 years) had the highest SARI-associated influenza hospitalization rates, consistent with trends identified in international literature, particularly those from developed countries with temperate climates.^10^,^72^–^75^

A high rate of influenza-related hospitalizations and low vaccine uptake (6%) in young children (6 months to 4 years) from SHIVERS led the NZ government to change vaccination policy by extending free influenza vaccination to those in this age group who have been hospitalized or have a history of significant respiratory illness.^76^

SARI surveillance is likely to underestimate the true burden of severe influenza resulting in hospitalization. Some patients will present with non-respiratory symptoms or respiratory disease that does not meet the SARI case definition, or stay briefly in emergency department.^63^,^66^ SHIVERS has begun to address this gap; a pilot study in 2012 testing persons with respiratory disease who did not meet the SARI case definition showed that a small proportion (6%) of non-SARI patients were positive for influenza viruses, compared with 18% of SARI cases (manuscript in preparation). Future work to expand the case definition to all acute hospital admissions in a sample of very young children will further expand our knowledge of the burden of influenza in this important group potentially protected by maternal immunization. Additionally, SARI and associated influenza cases will be linked to the hospital discharge data to determine the accuracy and validity of the discharge data by determining proportions of the principal discharge diagnosis code categories that are SARI and influenza cases. This will help inform modelling studies of ICD-coded data and help provide some validation of these with laboratory-confirmed data.

SARI surveillance is also likely to grossly undercount the actual number of influenza-associated deaths because only a minority of influenza-related SARI deaths are correctly diagnosed, tested and recorded as such. Additional influenza deaths resulting from secondary bacterial infections and exacerbation of pre-existing chronic conditions and atypical clinical presentations are not captured.^77^ This limitation presents a challenge in accurately measuring influenza-related mortality. Future work on statistical modelling may allow for indirect estimation of ‘excess’ mortality attributable to influenza in those broad categories such as pneumonia, respiratory or circulatory deaths during influenza seasons.^78^

Secondly, the disease burden of moderate influenza is estimated using data from SHIVERS ILI surveillance. Our findings from the 2013 season (29 April to 29 September) showed that the ILI-associated influenza consultation rate was about 14 times higher than SARI-associated influenza hospitalization rate (manuscript in preparation). Additionally, ILI-associated influenza consultations and SARI-associated influenza hospitalizations showed contrasting socio-demographic patterns: higher rates of ILI-associated influenza consultations were shown in preschoolers (aged 1–4 years), school-age children and adults (<65 years), those of Asian ethnicity and those from least deprived socio-economic status (SES) groups. This was a different picture from SARI surveillance where SARI-associated influenza hospitalizations were more frequent in the very young (under 1 year), the elderly, Māori and Pacific peoples and those from most deprived SES groups.^79^ These results provided insights into the interplay between healthcare access opportunities and related health-seeking behaviours and the differential effect of the predominant strains on various age groups.

Thirdly, the disease burden of mild influenza not requiring medical consultation (e.g. school/work-related absenteeism) and influenza infection (symptomatic/asymptomatic) will be estimated from the SHIVERS serosurvey. Additionally, we will also conduct severity assessment using true numbers of infections as the denominator to calculate case/fatality and case/hospitalization ratios. Furthermore, the data on influenza disease burden will allow us to estimate direct medical costs and indirect societal costs (e.g. loss of productivity, loss of earning and loss of life) for the study population and subpopulations.^80^,^81^

### Aetiology

Preliminary results in 2013 identified an under-recognized burden of non-influenza respiratory viruses, particularly RSV and rhinoviruses, in SARI and ILI cases in NZ as we have never had active population-based study on these viruses previously although substantial burden of RSV and rhinovirus has been described elsewhere.^82^,^83^ ILI-associated consultations and SARI-associated hospitalizations for RSV and rhinovirus show different socio-demographic patterns (age, ethnicity and SES) from that of influenza. For example, both ILI- and SARI-associated RSV incidences were similar – high rates for very young (<1 year and 1–4 years) followed by elderly (≥65 years). This presented a very different age-specific incidence profile from that of influenza (indicated above). This result, together with subsequent multiyear surveillance data, may provide insights on differential effects of various respiratory viruses on the age distribution of the host and disease severity.

### Vaccine effectiveness

SHIVERS surveillance platforms provided a systematic opportunity to estimate VE for the prevention of general practice visits and hospitalizations for RT-PCR-confirmed influenza from the same population in the same influenza season.^40^,^41^ A case test-negative control design is used to estimate annual propensity-adjusted vaccine effectiveness in both hospital and community settings. The data in 2013 showed moderate effectiveness of influenza vaccine against medically attended and hospitalized influenza in NZ with 56% (95% CI 34,70) against influenza presenting to general practice and 52% (95% CI 32,66) protection against laboratory-confirmed influenza hospitalization.^41^

### Immune responses

SHIVERS surveillance platforms also provided sera and whole-blood samples during acute and convalescent phases of infection to study humoral and cellular immune responses from a subset of severe (*N *= 39) and moderate (*N *= 29) influenza cases in 2013. With these samples, and using a combination of serological and immunological assays, we were able to (a) estimate the relative contribution of early adaptive and cellular immune responses to disease severity; (b) identify differences in the immune profiles between these diseases groups; and (c) identify immunological correlates of disease severity in subpopulations. Data acquired so far indicate that SARI cases may experience a more robust immunologic response during infection (i.e. greater increases in HA- and NA-specific antibody titres as well as magnitude of T-cell response). The ability to parse out immunological differences between severe and moderate influenza cases in this pilot cohort highlights the value of adding the active immunology study to the SHIVERS platforms.

### Risk factors

As NZ has a well-characterized socio-demographic distribution (age, sex, ethnicity, deprivation) from population census data, socio-demographic risk factors can be characterized quite easily. SHIVERS will use the results obtained from hospital and sentinel general practice surveillance to disentangle the effect of ethnic and socio-economic gradients.

For other more specific risk factors (e.g. host factors such as comorbidities, and environmental factors such as housing conditions), there are limited data available on their distribution in the population. Consequently, it is difficult to assess the importance of the risk factor data collected. There are several comparison/control groups such as hospital-based control populations without respiratory illness, serosurvey participants as a control group and SARI/ILI test-negative controls. These controls could be compared with SARI/ILI cases to estimate the importance of specific risk factors for influenza infection and related severe/moderate diseases including socio-economic, underlying medical conditions, health intervention, health service utilization, and environmental and behavioural factors.

## International applications

We have established active, prospective, population-based surveillance systems for a wide range of respiratory disease presentations and designed a portfolio of influenza studies based on these platforms. The SHIVERS results during the first 2 years (2012–2013) provided scientific evidence to support change to NZ's vaccination policy for young children due to high SARI-associated influenza hospitalizations in these children; contribute to the finalization of the World Health Organization's SARI case definition for global influenza surveillance; and contribute in part to vaccine strain selection with vaccine effectiveness assessment in the prevention of general practice visits and hospitalizations for laboratory-confirmed influenza. In the next 3 years (2014–2016), this project will continue to help us to understand (1) the burden of influenza infection including symptomatic/asymptomatic infection and mild disease not requiring medical consultation; (2) influenza risk that considers organism, host and environmental factors; (3) the impact of viral–viral and viral–bacteria co-infections on clinical disease and severity; and (4) the nature of responding adaptive immune responses in determining individual's risk of acquiring influenza virus infection.

Over 5 years, we hope this project will shed more light on the burden of influenza and other respiratory pathogens in our study population and subgroups and estimate key epidemiologic parameters such as relative rates of infection, clinical disease, general practice visits and hospitalizations as well as risk factors for illness, effectiveness of vaccination, mechanisms of immunity and monitoring for new influenza viruses with pandemic potential such as A(H7N9) and other emerging viruses (e.g. MERS-CoV) and provide a framework for timely assessment of severity which is essential in an event of emergence of these pathogens.

The platforms established here are relevant not only for New Zealand policy, but also for the region and the world. It will provide robust systematic virologic, epidemiologic and vaccine effectiveness data on circulating pandemic or seasonal influenza viruses at a time when circulation in the Northern Hemisphere is low. This could provide valuable information on all emergent respiratory pathogens that have some winter seasonality. Moreover, the data elements on a range of disease severities collected in the same population at the same time will generate epidemiologic parameters that maybe broadly generalizable and translatable to similar developed, southern and northern temperate countries worldwide. This will help enormously to better understand more basic surveillance data and to extrapolate those data in models to plan and predict influenza behaviour, generate burden estimates, model the impact of seasonal influenza vaccination to support more global use and better prepare for the next pandemic.

In summary, SHIVERS is expected to provide extensive data to guide improved methods for disease surveillance; improve clinical case management, early detection and optimization of laboratory diagnosis; inform vaccine strain selection and vaccine development; guide targeted vaccination strategies for population and subgroups; understand host immune responses and identify better immune diagnostic markers.

## Funding

The SHIVERS (Southern Hemisphere Influenza and Vaccine Effectiveness Research and Surveillance) project is funded by US Department of Health and Human Services, Centers for Disease Control and Prevention (CDC) (1U01IP000480-01). The project is a 5-year research cooperative agreement between ESR and US CDC's National Center for Immunization and Respiratory Diseases Influenza Division.

## Authors’ contributions

Q. Sue Huang: principal investigator of SHIVERS; Nikki Turner: lead objective 2 (vaccine effectiveness); Michael G. Baker: lead objectives 3 (interaction) and 7 (risk factors); Deborah A. Williamson: coordinate objective 1 (hospital surveillance); Conroy Wong: co-lead objective 1 (hospital surveillance); Richard Webby: lead objective 8 (immunology); Marc-Alain Widdowson: CDC's project officer of SHIVERS. All authors participated in designing and implementing the SHIVERS project, interpreting the results, and developing and revising the manuscript critically for intellectual content. All authors have given final approval of the version to be published. The findings and conclusions in this article are those of the authors and do not necessarily represent the official position of the US Centers for Disease Control and Prevention (US CDC), the Institute of Environmental Science and Research (ESR) and other collaborating organizations.

## Appendix 1 The SHIVERS investigation team (listed in an alphabetic order)

Debbie Aley, Don Bandaranayake, Ange Bissielo, Kirstin Davey, Jazmin Duque, Cameron C. Grant, Diane Gross, Shirley Lawrence, Graham Mackereth, Barbara McArdle, Colin McArthur, Nevil Pierse, Sarah Radke, Sally Roberts, Ruth Seeds, Susan Taylor, Paul Thomas, Mark Thompson, Angela Todd, Adrian Trenholme, Tim Wood, Sook-San Wong.
